# D5 dopamine receptors control glutamatergic AMPA transmission between the motor cortex and subthalamic nucleus

**DOI:** 10.1038/s41598-018-27195-6

**Published:** 2018-06-11

**Authors:** Lionel Froux, Morgane Le Bon-Jego, Cristina Miguelez, Elisabeth Normand, Stephanie Morin, Stéphanie Fioramonti, Massimo Barresi, Andreas Frick, Jerome Baufreton, Anne Taupignon

**Affiliations:** 1 Univ. de Bordeaux, Institut des Maladies Neurodegeneratives, UMR 5293, F-33000 Bordeaux, France; 2 CNRS, Institut des Maladies Neurodegeneratives, UMR 5293, F-33000 Bordeaux, France; 3 Department of Pharmacology, Faculty of Medicine and Dentistry, University of the Basque Country (UPV/EHU), 48940 Leioa, Spain; 4 Department of Pharmacology, Faculty of Pharmacy, University of the Basque Country (UPV/EHU), 01006 Vitoria-Gasteiz, Spain; 5 Univ. de Bordeaux, Institut Interdisciplinaire des Neurosciences, UMR 5297, F-33000 Bordeaux, France; 6 CNRS, Institut Interdisciplinaire des Neurosciences, UMR 5297, F-33000 Bordeaux, France; 7 INSERM, Neurocentre Magendie, Physiopathologie de la plasticité neuronale, U1215, 33077 Bordeaux cedex, France; 8 Univ. Bordeaux, Neurocentre Magendie, Physiopathologie de la plasticité neuronale, U1215, 33077 Bordeaux, cedex France

## Abstract

Corticofugal fibers target the subthalamic nucleus (STN), a component nucleus of the basal ganglia, in addition to the striatum, their main input. The cortico-subthalamic, or hyperdirect, pathway, is thought to supplement the cortico-striatal pathways in order to interrupt/change planned actions. To explore the previously unknown properties of the neurons that project to the STN, retrograde and anterograde tools were used to specifically identify them in the motor cortex and selectively stimulate their synapses in the STN. The cortico-subthalamic neurons exhibited very little sag and fired an initial doublet followed by non-adapting action potentials. In the STN, AMPA/kainate synaptic currents had a voltage-dependent conductance, indicative of GluA2-lacking receptors and were partly inhibited by Naspm. AMPA transmission displayed short-term depression, with the exception of a limited bandpass in the 5 to 15 Hz range. AMPA synaptic currents were negatively controlled by dopamine D5 receptors. The reduction in synaptic strength was due to postsynaptic D5 receptors, mediated by a PKA-dependent pathway, but did not involve a modified rectification index. Our data indicated that dopamine, through post-synaptic D5 receptors, limited the cortical drive onto STN neurons in the normal brain.

## Introduction

Unexpected events often occur in everyday life, requiring interruptions and changes to ongoing behavior. Studies in humans in a variety of tasks have suggested that slowing, changing, or interrupting ongoing behaviors are implemented by an input from the cortex to the subthalamic nucleus (STN) (for review^1^). This has also been suggested for rodents^2,3^, but whether the stop signal involved the cortex to STN connection, as postulated from human experiments, has not been established^4^.

The STN is a component nucleus of the basal ganglia, a collection of interconnected, subcortical nuclei that process cortical information, in order to promote contextually appropriate actions enacted by cortical and brainstem motor centers. Excitatory corticofugal fibers target neurons in the striatum that form the so-called direct and indirect basal ganglia pathways. A subset of neurons in various cortexes, including the motor cortex, targets the STN, the sole small excitatory (glutamatergic), nucleus of the basal ganglia, forming the cortico-subthalamic (Cx-STN) connection, often called the ‘hyperdirect’ pathway in basal ganglia studies^5–8^. Information transfer along this pathway seems faster and more broadly distributed among the basal ganglia output stations than in the two others^9–11^.

Task-irrelevant global motor suppression has also been attributed to the Cx-STN connection^12^. Indeed, a well-established feature of Parkinsonian states, characterized by motor symptoms, including akinesia, is a change in STN firing, associated with an increase in beta-band oscillations of the local field potential^13–15^, with cortex activity then leading the STN^14,16^. The membrane properties of STN neurons, together with timely inhibitions and excitations due to neurons in the indirect and Cx-STN pathways, respectively, are thought to underlie the synchronized pathological oscillations^17,18^. In humans, an increased functional connectivity has been reported between some cortical areas, including the motor cortex and the STN^19,20^. However, structural connectivity may be diminished, since a partial loss of the hyperdirect motor Cx-STN projection has been found in MPTP-treated parkinsonian monkeys^21^. Finally, in Parkinson patients, high-frequency stimulation of the STN provides a significant improvement in cardinal motor symptoms. Data from rodents and humans support the hypothesis that the Cx-STN connection contributes to these beneficial motor effects^22–24^’.

All these data have prompted recent re-evaluations of Cx-STN hyperdirect pathway functions in healthy and diseased humans and rodents^1,25–27^.

Nevertheless, the properties of the cortical neurons that project to the STN are unknown, as most cortical fibers projecting to the STN are severed from somas in brain slices and electrical stimuli activate axons belonging to other nuclei. Another unknown is whether dopamine controls the Cx-STN synapses, as it controls cortico-striatal synapses. D5 dopamine receptors are expressed in cortical and subcortical structures^28,29^. Their role is rarely investigated due to the lack of subtype-specific ligands in the D1 family. In the STN, they strengthen oscillatory burst firing. They are expressed in the soma and dendritic processes at asymmetric synapses, suggesting that they may control glutamatergic afferents^30^. In these experiments, a retrograde labelling strategy was used to identify Cx-STN neurons in living brain slices. An optogenetic approach was used to isolate cortical afferents and investigate the properties of AMPA Cx-STN transmission. This elucidated, for the first time, the specific neuronal properties of the pathway and revealed that it was controlled by post-synaptic dopamine D5 receptors in the physiological state.

## Results

### Properties of Cx-STN neurons in the motor cortex

**Figure 1:**
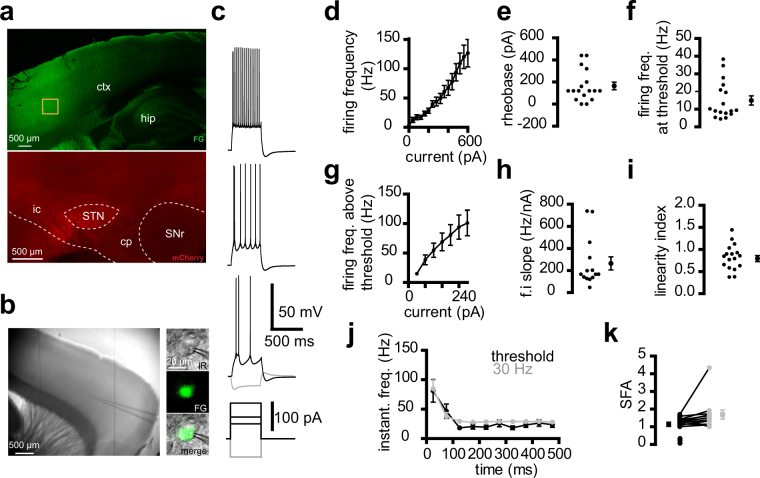
Properties of cortico-subthalamic neurons. (**a**) Confocal images of the cortex (top) after an injection of Fluoro-Gold (FG)retrograde tracer in the STN. To check the location of the injection site (bottom), the FG solution was mixed with a solution of anterograde virus expressing mCherry that labelled neurons at the injection site. FG and mCherry were detected by immunocytochemistry in a 350 μm slice used for electrophysiology. The orange box on the cortex indicates a high-density zone of FG-labelled neurons. Ctx, cortex; hip, hippocampus; ic, internal capsule; cp, cerebral peduncle; SNr, substantia nigra reticulata. (**b)** Infrared view of a living slice (left) and an FG-positive neuron at high magnification (right). (**c)** Example of the electrical properties of a retrograde-labelled cortico-subthalamic neuron. (**d**–**i)** Input-output relationship of a sample of 17 cortico-subthalamic neurons. (**d)** Mean firing frequency-current relationship. (**e**) Rheobase. (**f)** Firing frequency at threshold. (**g)** Mean firing frequency-current relationship above threshold. The current values represent the amplitude of step stimuli minus the amplitude of the first action potential-evoking step. (**h)** Firing frequency–current (f.i) slope. (**i)** Linearity index of the firing frequency–current relationship. e,f,g,h,i, left: individual values, right: mean ± sem. (**j**,**k)** Spike frequency over time in the same sample. (**j)** Instantaneous firing rate versus time (time was divided into 50 ms bins; times shown are the center of each bin) and (**k**) Spike firing adaptation, with black and grey symbols for values at threshold and 30 Hz, respectively. Three neurons with firing frequencies close to 30 Hz at threshold and those with above threshold values much higher than 30 Hz were not included in the 30 Hz data. (**c**–**k)** Saline was supplemented with 20 μM DNQX, 50 μM APV, 50 μM picrotoxin, and 1 μM CGP 55845.

Cx-STN neurons form a small subpopulation of projection neurons in the cortex^6^. Their electrical properties are unknown. A discrete population of Cx-STN neurons was labeled and recorded in living slices. A fluorescent retrograde tracer was injected into the STN of young adult mice and acute brain slices were prepared 1 to 3 weeks later. Injection of Fluoro-Gold retrograde tracer within the boundaries of the STN resulted in discrete labelling in the motor cortex (Fig. 1a). Fluoro-Gold was also detected in neuron cell bodies in living slices (Fig. 1b). The fluorescent Cx-STN neurons showed a specific intrinsic property, a minimal hyperpolarization-activated sag, and their firing was characterized by an initial doublet, as well as virtually no adaptation over time (Fig. 1c). They had a mean resting membrane potential of −71.1 ± 1.5 mV (n = 15) and a membrane resistance of 103 ± 12 mΩ (n = 17). The sag index was 4.0 ± 0.5% (n = 17), with minimum and maximum values of 0.9% and 7.3%, respectively. Note that 2 neurons in the sample were spontaneously active, with firing frequencies of 4.46 and 5.37 Hz, respectively, at rest, but their sag values were in the same range as those of the other 15 neurons. Firing frequency exceeded 100 Hz when the neurons were strongly depolarized, but mean firing frequency at threshold was around 15 Hz (14.9 ± 2.5 Hz, n = 17, range 5–38 Hz), whereas the mean rheobase was 164 ± 34 pA (n = 17) (Fig. 1d–f). Firing frequency above the threshold was characterized by an input-output (f.i slope) of 347 ± 78 Hz/nA and firing rates gradually rose less steeply with increasing current injection (linearity index: 0.82 ± 0.07, n = 17) (Fig. 1g–i). Firing over time exhibited a high instantaneous frequency, due to the initial doublet of action potentials, and remained stable after the first 100 ms (Fig. 1j). There was little spike firing adaptation (SFA) at threshold, as well as at 30 Hz (SFA values close to 1) (Fig. 1k).

### Synaptic transmission from the motor cortex to the STN involves GluA2-lacking AMPA receptors

**Figure 2:**
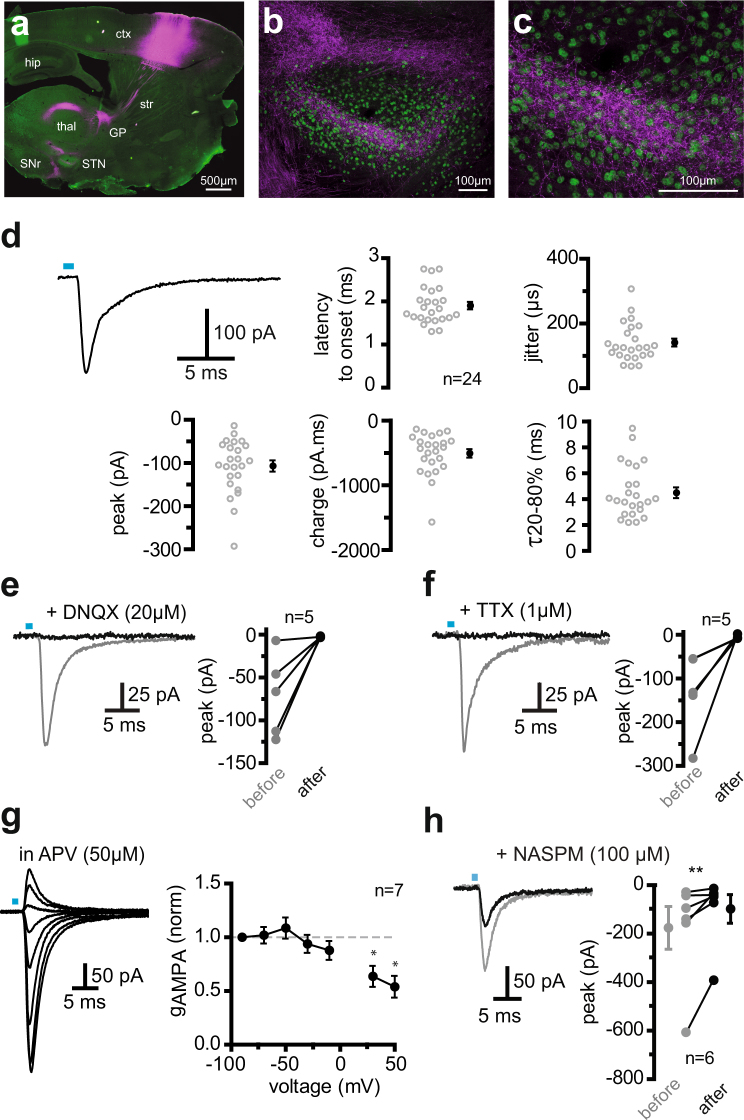
Optical activation of corticofugal fibers in the STN results in action potential-dependent AMPA synaptic transmission (**a**) Expression of mCherry following transfection of a ChR2-mCherry viral solution in the motor cortex. (**b**,**c**) mCherry-positive corticofugal fibers in the STN. The STN boundaries are delineated by the STN neuron-specific FoxP2 marker (in green). (**d**) Example of the current evoked by a 1 ms flash and descriptive parameters of the inward currents measured in a sample of 24 subthalamic neurons. Current peak amplitude and charge, onset latency, time required to decay from 80 to 20% of peak value, and jitter are reported as individual values (left) and mean ± sem (right). Flash duration and luminance ranges were 0.4 to 1 ms and 0.55 to 1.15 mW.mm^−2^. (**e**,**f**) The light-induced inward currents were fully blocked by the specific AMPA/kainate receptor antagonist, DNQX (20 µM), or the sodium channel inhibitor, TTX (1 µM). The gray and black traces illustrate currents photo-evoked in controls and in the continued presence of the drugs, respectively. The graphs to the right of each recording display the summary data of current values before and after drug application. (**g**) Voltage-dependence and conductance of pharmacologically-isolated AMPA receptor EPSCs. Left: Representative traces of photo-induced EPSCs in the presence of the specific NMDA receptor antagonist, APV (50 µM). Holding voltage was changed from −90 to +50 mV in 20 mV steps. Right: Plot showing chord conductance against voltage. Conductance was significantly different from 1 at +30 and +50 mV, indicating rectifying AMPA receptors. (**h**) Naspm reduced the EPSC magnitude, indicating Ca^2+^-permeant, GluA2-lacking, as well as GluA2-containing, AMPA receptors. ^*^Significantly different from the theoretical median = 1, two-tailed Wilcoxon signed rank test, p = 0.0313 for both +30 mV and +50 mV. ^**^Indicates significant changes at p = 0.0156, one-tailed Wilcoxon matched-pairs signed rank test.

Corticofugal fibers target the STN and stimulate STN neurons in animals. To investigate the synaptic properties of the Cx-STN synapses, the stimulation must be specific to the cortical afferent fibers and avoid activating the afferent fibers of any other afferent nuclei, such as thalamus or brain stem nuclei^31–33^. This was achieved using anterograde transfection of neurons in the cortex. Injections of ChR2-mCherry-expressing adenovirus were targeted at the motor cortex, resulting in robust expression in cells, as well as in corticofugal fibers within the striatum, thalamus, internal capsule, and *zona incerta* (Fig. 2a).

Within the STN, delineated by the STN neuron-specific FoxP2 marker^33^, mCherry fluorescence was observed in a bundle-like area in the lateral part of the nucleus (Fig. 2b–c). Cx-STN fibers were activated using blue light pulses delivered by an optical fiber placed over the internal capsule or STN. Brief laser flashes (0.4 to 1 ms) produced inward currents in the STN neurons located within the dense bundle of fluorescent fibers (Fig. 2d). No response was evoked in neurons outside the fluorescent area of the STN or when viral DNA included the sequence coding for a reporter alone, rather than for the fusion protein ChR2-reporter (EYFP, n = 4; mCherry, n = 4). In a sample of 24 neurons, light-evoked currents ranged from −14 to −293 pA, with a mean amplitude of 107 ± 13 pA and a mean charge close to −500 pA.ms. The latency between flashes and evoked currents was always greater than 1 ms, with a mean of 1.90 ± 0.08 ms, and the mean jitter value was 140 ± 12 μs, consistent with monosynaptic transmission (Fig. 2d). The AMPA/kainate receptor antagonist, DNQX, was applied to characterize the laser-evoked currents (Fig. 2e). This blocked the currents completely in all 5 cells tested, demonstrating that glutamate was released on photostimulation and that ionotropic glutamate AMPA/kainate receptors were involved. In similar conditions, a selective antagonist of AMPA receptors GYKI53655 (50 μM) inhibited currents, indicating minimal contribution of kainate receptors^34^. To assess whether glutamate release was initiated by action-potential-dependent processes, robust laser-evoked Cx-STN responses were recorded in STN neurons and then 1 µM TTX was applied to block voltage-dependent sodium channels. TTX completely abolished the responses in all 5 neurons, indicating that the glutamate release was action potential-dependent (Fig. 2f). Light-induced currents were thus AMPA-mediated excitatory post-synaptic currents (EPSCs).

A study of nucleated patches from the STN neurons of immature rats suggested that AMPA receptors in STN neurons lacked GluA2 subunits^35^. I-V curves of photo-induced AMPA synaptic currents reversed close to 0 mV and displayed rectification (Supplemental Fig. S1). Normalized chord conductances were not significantly different from 1 in the negative potential range, but differed significantly from 1 at depolarized potentials (Fig. 2g). The synthetic analogue of Joro spider toxin, 1-naphthyl acetyl spermine (Naspm,100 μM), which selectively binds to GluA2-lacking AMPA receptors, was active in all neurons tested, reducing the average EPSC magnitude by 44 ± 0.09% (Fig. 2h). Thus, GluA2-lacking AMPA receptors are a specific feature of Cx-STN synapses in mature rodent brains.

### Short-term plastic properties of motor Cx-STN synapses

**Figure 3:**
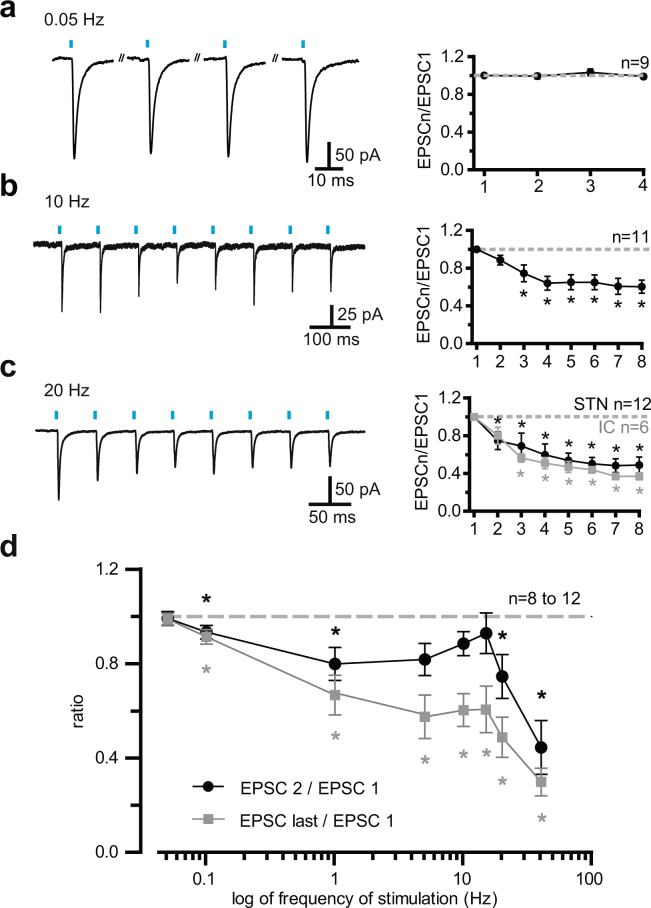
Short-term plasticity of AMPA/kainate cortico-subthalamic transmission at stimulation frequencies between 0.05 and 40 Hz (**a**,**c**) Examples of EPSCs obtained at light frequencies of 0.05, 10, and 20 Hz (left) and group synaptic dynamics data (right). Mean response amplitudes were normalized to the first responses and plotted as a function of stimulus number within trains. The gray symbols in C depict the values obtained by flashing the internal capsule (and not the STN). The stimuli trains contained 8 photo-stimuli, except those at 0.05 Hz, which comprised only four photo-stimuli. (**d**) Summary graph of the filter properties of the cortico-subthalamic synapses. The black line and symbols display the ratios of paired pulse values, i.e. peak values of the second to the first EPSC in a train. The gray plot shows the ratio of the last to the first EPSC in a train (the 8th EPSC was the last, except for the tests at 0.05 and 0.1 Hz, where the ratio of the fourth to the first EPSC was calculated). As paired-pulse ratios exhibited no change in the 5 to 15 Hz range, a limited band-pass frequency was assumed. ^*^Values significantly different from 1, α = 0.05, two-tailed Wilcoxon signed rank test.

Since short light flashes activated action potential-dependent AMPA synaptic transmission, the next step was to investigate the dynamic properties of the AMPA currents in the STN (Fig. 3). Layer 5 pyramidal neurons in the motor cortex fired with a fidelity of 100% on photoactivation by trains of 8 flashes at frequencies up to 40 Hz (Supplementary Fig. S2). Furthermore, as shown in Fig. 1, the firing frequency at threshold of identified cortical neurons projecting on the STN was close to 10 Hz and always below 40 Hz. Thus, to reproduce the possible range of firing in the motor cortex, we used trains of flashes in the 0.05 to 40 Hz range. Figure 3a–c shows examples of AMPA/kainate EPSCs induced in STN neurons by trains of flashes at 0.05, 10, and 20 Hz. The only stimulation frequency that did not produce any significant change in the EPSC amplitude was 0.05 Hz. Apart from the first two flashes, 10 Hz yielded a depression. At a flash frequency of 20 Hz, all the EPCSs decreased. The group data (Fig. 3d) recapitulates these contrasting properties. Whereas a frequency of 0.05 Hz evoked constant EPSCs, at stimulation frequencies of 0.1 and 1 Hz, as well as 20 and 40 Hz, irrespective of their rank in the trains, EPSC amplitude always decreased significantly. In contrast, EPSCs evoked in the 5 to 15 Hz range displayed a ratio of 1 for the first two stimuli (EPSC2/EPSC1), followed by a decrease in the other EPSCs evoked by subsequent flashes. The group with a PPR of 1 comprised neurons that displayed a small potentiation or depression. Thus, 2 neurons out of the 11-neuron sample displayed short-term potentiation at 10 Hz, with a mean PPR of 1.10. There was, therefore, a small window with faithful transmission of the first two stimuli in a train in the 5 to 15 Hz range, a frequency domain close to the mean firing frequency of Cx-STN neurons at threshold. It was noteworthy that flashing the internal capsule produced the same short-term depression as flashing the STN (Fig. 3c, and Supplemental Fig. S3, grey symbols and lines).

### Dopamine D1/D5 receptor activation reduced the synaptic strength at Cx-STN synapses

Dopamine depletion has been shown to change the structural connectivity between the cortex and STN in non-human primates^21^, as well as rodents^26^, and functional connectivity in human studies^19,20^. These results raised the possibility that dopamine controlled Cx-STN synaptic strength, in addition to that of cortico-striatal synapses. Since we demonstrated that D5 receptors in the STN were expressed in or close to excitatory synapses^30^, we tested the hypothesis that Cx-STN transmission was controlled by dopamine acting on D5 receptors.

**Figure 4:**
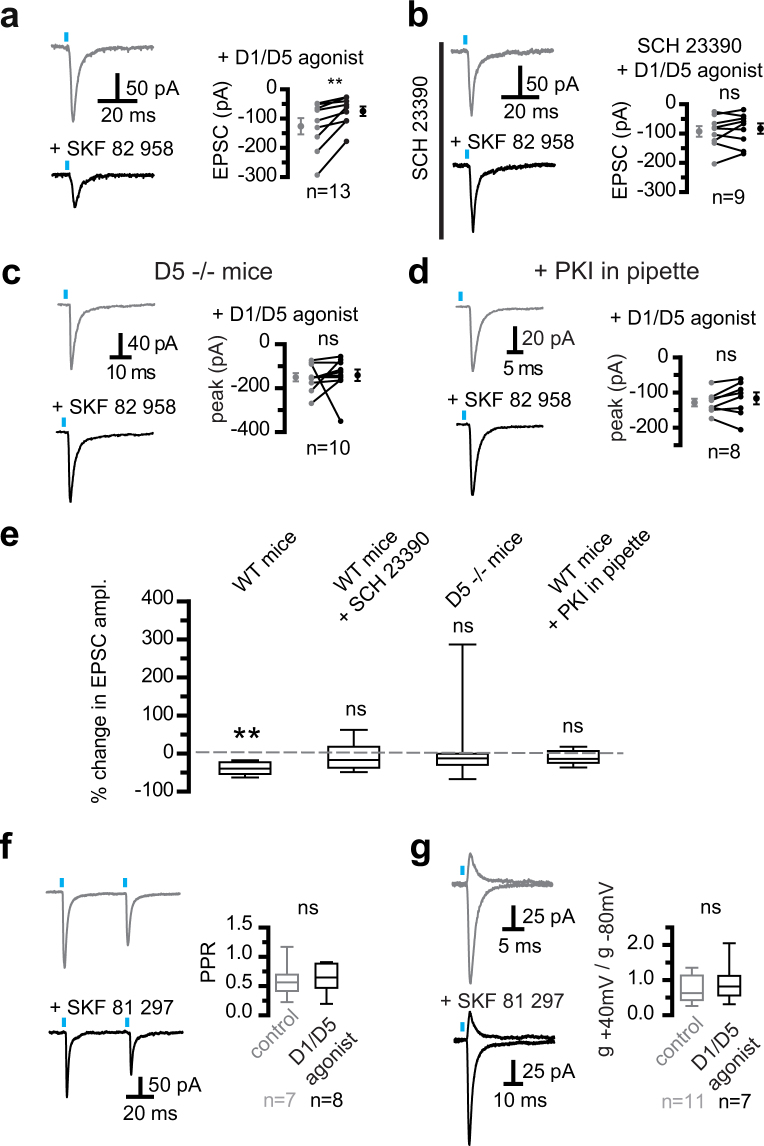
D5 dopamine receptors reduced AMPA/kainate synaptic strength. (**a**) Activating D1/D5 receptors reduced cortico-subthalamic AMPA/kainate EPSCs in subthalamic neurons. Illustration of the action of the D1/D5 agonist, SKF 82958 (5 µM). D1/D5 agonists refer to SKF 82958, SKF 81297, or SKF 38393, at 2–5 µM. The 3 dopaminergic agonists were used to neutralize the differences in the potency of the drugs or their affinity for D5 or D1 receptors, as reported in the literature. (**b**) The continued presence of SCH 23390 (10 µM) prevented the action of SKF 82958 (5 µM). (**c**) Perfusion of 5 µM D1/D5 agonists had no significant effect in slices from D5^−/−^ mice. (**d**) D1/D5 agonists (5 µM) were no longer active in the neuron sample tested with a peptide inhibitor (PKI, 20 µM in pipette solution) to intracellularly inhibit protein kinase A. In (**a)** to (**d)**, traces show the currents recorded immediately prior to (top) and 10 min after agonist perfusion (bottom), and group data show individual paired values as well as mean ± sem. Two-tailed Wilcoxon matched-pairs signed rank tests were used; α was 0.05; **indicates significant changes at p = 0.0039; ns, not significant. (**e**) Box plot summary of the changes in EPSC amplitude obtained under the conditions depicted in a to d. The boxes present the distribution with the median as a central line. The hinges and edges display the 25^th^ and 75^th^ percentiles, whereas the “whiskers” display the minimal and maximal values. Action of D1/D5 agonists only reached significance in wild-type mice and not in any other 3 conditions. A Wilcoxon signed rank test was used to test a null hypothesis (no action of D1/D5 agonists). α = 0.05; **significant changes at p = 0.0039; ns, not significant. (**f**) No change in photo-induced paired pulse ratio (PPR) when D1/D5 receptors were activated. (**g**) The conductance ratio at −80 mV and +40 mV is the rectification index. It did not change in neurons bathed in D1/D5 agonists for 10–60 min. Left, representative traces of EPSCs photo-evoked at −80 mV and +40 mV in control (top) and 5 μM SKF 81 297 (bottom); right, group data showing the ratio of chord conductance at +40 mv to that at −80 mV. The reversal potential of the synaptic response in the presence of D1/D5 agonists was measured in 3 neurons, where a full I.V curve was established. Examples in (**f**) and (**g**) depict different neurons and group data report EPSCs obtained in saline alone or supplemented with 5 µM D1/D5 agonist for 10 to 60 min.

We used 3 dopaminergic agonists (SKF 82958, SKF 81297 or SKF 38393, 2–5 µM, referred to collectively as ‘D1/D5 agonists’) to neutralize any possible difference in the affinity or potency of the drugs for D5 or D1 receptors, as reported for recombinant D1 and D5 receptors^36–38^. Unexpectedly, and irrespective of the agonist, D1/D5 activation reduced Cx-STN transmission in all 13 neurons tested (Fig. 4a). This was truly a D1/D5-mediated action, since it was occluded when a D1/D5 agonist was co-applied with the D1/D5 antagonist, SCH23390 (10 µM) (Fig. 4b,e). D1/D5 agonists significantly reduced the EPSC amplitude from −126 ± 27 pA to −75 ± 16 pA (p = 0.0039, n = 13, two-tailed Wilcoxon matched-pairs signed rank test). The mean reduction in EPSC amplitude was −38 ± 5%. Accordingly, the EPSC charge was reduced from −643 ± 145 pA.ms to 407 ± 89 pA.ms (−37 ± 7%, p = 0.0039, n = 13).In the continued presence of SCH23390, D1/D5 agonists no longer had any significant action. Mean control and test amplitude values were −94 ± 18 pA and −84 ± 17  pA, respectively (p = 0.30, n = 9, Wilcoxon matched-pairs signed-rank test). The charge also remained unchanged (control: 297 ± 43 pA.ms; test: 264 ± 47 pA.ms). At the concentrations required to act on neurons in brain slices, D1/D5 family receptor agonists do not discriminate between D1 and D5 receptors, although they show some specificity on recombinant receptors expressed in heterologous systems^36–38^ and cell preparations^39^. Our results may, therefore, be due to the activation of either D1 or D5 receptors. However, there is no experimental evidence of D1 receptor expression in the STN, whereas D5 receptors have been identified^28,30^. To test the assumption that the inhibitory action of the D1/D5 family agonists was mediated by D5 receptors, D5^−/−^ mice^40^, bearing a null mutation of the D5 receptor, were infected with the same adeno-viral expression vector as wild-type mice. The D1/D5 agonists did not produce a significant change in the EPSC amplitude or charge (Wilcoxon matched-pairs signed-rank test, n = 10, p = 0.10 and p = 0.13 for amplitude and charge, respectively) (Fig. 4c). This suggested that D5 receptors were involved in the inhibitory action of D1/D5 agonists on AMPA/kainate EPSCs. It should be noted though that there was variation in the dataset. Activation of recombinant D5 receptors is known to consistently produce elevation of cAMP, when expressed in heterologous models, such as HEK293^36–39^. Accordingly, the action of D5 receptors on burst firing in the STN was mediated by a cAMP/PKA pathway^30^. The protein kinase A peptide inhibitor, PKA inhibitor fragment (6–22) amide, referred to as PKI, was used in the pipette solution to block the cAMP/PKA transduction pathway before testing the action of the D1/D5 receptor agonists (Fig. 4d). When the pipette solution was supplemented with 20 μM PKI, the absence of significant changes in the light-induced EPSCs indicated that the D1/D5 agonists were inactive. The group data values were not significantly different: −129 ± 11 pA (mean control values) and −117 ± 17 pA (mean test values), (p = 0.31, Wilcoxon matched pairs signed-rank test, n = 8). Furthermore, no significant change was observed in the EPSC paired-pulse ratio (PPR) when 20 Hz flashes were applied to neurons kept in saline or bathed in a D1/D5 agonist (5 µM) for 10 to 60 min. The PPR (peak current ratio of photo-induced EPSC_2_ and EPSC_1_) was similar in both conditions (Fig. 4f). PPR was 0.60 ± 0.11 (n = 7) in control and 0.65 ± 0.09 (n = 8) in D1/D5 agonist (p = 0.54, Mann and Whitney test for independent samples). Altogether, these experiments established that the D5 receptors were postsynaptic. In some neuron classes, changes in synaptic strength involve modifications in the subunit composition of AMPA receptors. The replacement of GluA2-lacking receptors by ones containing GluA2 results in a smaller EPSC amplitude at negative membrane potentials and an increased EPSC amplitude at depolarized potentials^41^. The next experiment tested whether D5 receptors triggered a switch in postsynaptic AMPA receptor subunit composition and, presumably, Ca^2+^ permeability. Rectification indexes were collected from neurons in saline alone and supplemented with 5 μM D1/D5 agonist for 10–60 min. EPSCs were measured at −80 and + 40 mV and the chord conductance ratio was calculated to obtain a rectification index (Fig. 4g). The rectification indexes were not significantly different, suggesting that GluA2 subunits were not incorporated into AMPA receptors at the Cx-STN synapses (mean control, 0.74 ± 0.12, n = 11, and mean D1/D5 agonists, 0.94 ± 0.21, n = 7, p = 0.46, Mann and Whitney test for independent samples).

## Discussion

The Cx-STN hyperdirect pathway is a monosynaptic pathway from the cortex to the STN, a component nucleus of the basal ganglia. Our results provide the first data on the presynaptic side of the pathway.

Cx-STN neurons project to the STN ipsi-laterally. They also project to many other structures, such as the striatum and thalamus, in addition to brainstem areas and the spinal cord^6,7^. As such, they belong to the broad class of pyramidal tract neurons^42^. Their only specific, intrinsic property is their hyperpolarization-activated sag, which is much smaller than that reported for the class as a whole^43,44^. Their firing is characterized by an initial doublet, followed by stable, non-accommodating, action potential firing, underlying a clearly biphasic, instantaneous spike frequency rate versus time. This property had not generally been reported for corticospinal neurons in the motor cortex^43,44^. It is important to determine whether the Cx-STN neurons belong to a subclass of pyramidal tract neurons and have specific intra-cortical circuit properties, since this will help to understand the function(s) of the Cx-STN pathway and the role of the projections of the Cx-STN neuron population to structures other than the STN.

The results presented in this paper provide the first experimental data on the short term plasticity of AMPA Cx-STN synapses. This approach reproduces the electrical protocols previously used to stimulate cortical afferents^45,46^, but has the unique property of being specific to corticofugal inputs: the synapses described here are truly Cx-STN synapses with no involvement of thalamus, brainstem, or midbrain components. Firstly, ChR2 was able to reliably fire pyramidal cell bodies up to 40 Hz. Secondly, light evoked action potential-dependent glutamate release. Thirdly, stimulating the internal capsule or Cx-STN terminals produced similar results. However, light-evoked synaptic responses may exhibit artificial synaptic depression, depending on the AAV expression vector serotype, as well as the stimulation technique, in a neuron-specific manner^47^. Since electrical stimulation of the vast majority of cortical areas connected to the STN, including the motor cortex, is impractical in slices^48^, one possible way of assessing the dynamics of motor Cx-STN responses will be to compare the properties of each excitatory afferent of the STN with the properties revealed by global electrical stimulation of all excitatory afferents. Finally, studying the responses of spontaneously firing neurons to trains of motor cx-STN EPSPs will reveal features specific to synaptic integration in the STN. For example, kainate receptors may contribute to EPSPs summation and affect the integration of diverse synaptic inputs since they do so in other brain structures^49^.

Dopamine is critical for the functions supported by the STN. It acts on the STN *via* pre- and post-synaptic receptors in the D1 and D2 families. To date, there is no evidence to support the expression of the D1 subtype in the STN of Rodents, although Primates express both D1 and D5 subtypes^50^. In the physiological state, Cx-STN transmission is likely to be reduced by the action of postsynaptic D5 receptors (this study) and presynaptic D2 receptors^51^, if the latter are on Cx-STN afferents, rather than other glutamatergic thalamic, brainstem, or midbrain afferents^31–33^. Estimates of DA affinities suggest that DA levels in the STN readily activate both receptors^52,53^. Thus, dopamine may limit the cortical drive onto STN neurons in the normal brain.

The indirect pathway afferents are also subject to negative control but by D2/D3 receptors only, since there is no evidence of receptors in the D1 family acting on these afferents^54^. Post-synaptic receptors in both families act on the firing properties of STN neurons. The D5 subtypes potentiate spontaneous and rebound burst firing, an action opposed by post-synaptic D2 receptors, which ultimately strengthen regular firing^30,55–57^. Besides, data obtained in slices from DA-deprived animals^58,59^ or, more recently^26^, are characterized by changes in sensitivity to dopamine and long-term structural changes, and therefore cannot be entirely inferred from acute data.

Despite widespread distribution in the brain^28,29^, D5 receptor signaling in neurons is still largely unknown. These data established that D5 receptor action on AMPA synaptic transmission involved PKA-dependent phosphorylation, since it was occluded by a PKA-specific inhibitor. Accordingly, cAMP/PKA signaling is the typical intracellular pathway of D5 receptors^36–38^, although other or complementary signaling pathways have been proposed for various cell types, including neurons^39^. However, in neurons, the ultimate result of PKA signaling is usually an increase in AMPA currents *via* phosphorylation of the GluA1 subunit, which promotes AMPA receptor insertion at extrasynaptic sites^60–62^. To our knowledge, the only example of AMPA current inhibition following D5 receptor activation was reported by^63^, see their Fig. 1b.

Changes in biophysical properties, either directly, *via* subunit composition, or receptor trafficking, may explain AMPA current inhibition. The phosphorylation state of AMPA receptor subunits by protein kinases/phosphatases is known to change synaptic strength bidirectionally. The reduction in AMPA current may then involve intermediates downstream from PKA^64,65^. AMPA receptor transmission may also be controlled by exchanging GluA2-containing and Ca^2+^-permeable GluA2-lacking receptors. GluA2-lacking receptors have been reported in the STN of immature rodents^35^. Our experiments revealed that GluA2-lacking receptors remained a feature of Cx-STN transmission in mature animals, but the rectification index was unchanged after D5 receptor activation. Receptor subunits are thus unlikely to have caused the change in AMPA current found here, though AMPA current was diminished and receptor composition shifted towards more abundant Ca^2+^-permeable GluA2-lacking receptors following dopamine depletion in mice^26^. Finally, auxiliary proteins (scaffold and cytoskeletal proteins, as well as AMPA-receptor interacting proteins and kinase anchor proteins) have been identified as regulators of AMPA receptor clustering, trafficking, and function. One auxiliary protein, GSG1L, has recently been identified as a negative regulator of AMPA receptors^66^ and might also be a target for D5 receptors.

Data obtained *in vivo* are equivocal: an increased coherence between the cortex and STN has been reported in Parkinson’s patients, as well as experimental models of the disease^14,16,67,68^, but long-term dopamine depletion attenuates the cortical innervation of the STN in primates^21^ and rodents^26^. At the basal ganglia circuit level, a decrease in Cx-STN transmission has been found in dopamine-deprived mice, whereas Cx-STN-induced heterosynaptic potentiation of inhibitory pallido-STN transmission was occluded in an NMDA-, as well as striato-pallidal-dependent manner, and the ratio of AMPAR to NMDAR currents was maintained^26,34^. In addition, Li *et al*. reported that, in awake, dopamine-deprived rodents, Cx-STN neurons in M1 were less active but fired more often in bursts, and had more coherent firing activities^23^. How these phenomena in the cortex and basal ganglia generate the increased coherence between the cortex and STN and/or the Parkinsonian-like symptoms has not yet been demonstrated.

The negative control of glutamate transmission is presumably partially maintained in dopamine-depleted states, due to the D5 receptors’ high intrinsic activity^69^. In Parkinson’s patients and experimental models of the disease, the strength of cortical input to the STN also presumably results from the balance between the possible lack of action of D2 receptors^51^ and the continued action of D5 receptors (present study), together with adaptive changes to continued dopamine deprivation^26,34^.

## Methods

### Animals

Procedures designed in agreement with the directive 86/609/EEC of the European Community Council were approved by the Comité d’Ethique en Experimentation Animale n°50 (agreement number: CE5012077-A). C57Bl6 and dopamine D5 receptor null mutant mice (D5^−/−^ mice)^40^ were used.

### Identification of Cx-STN neurons

We used a retrograde strategy with three types of tracers, a fluorescent dye and two viral expression vectors. The fluorescent dye Fluoro-Gold (aminostilbamidine, 2% in 0.9% NaCl) was bought from Invitrogen. A canine adenovirus (CAVDsREDII, 2.5 10^12^ IU/mL) was purchased from the Institut de Genetique Moleculaire de Montpellier^70^. A rabies-based recombinant virus (RabV, 1×10^6^ IU/ml) was also used. It was a glycoprotein deleted variant of the vaccine strain of rabies virus, SAD B19, incapable of trans-synaptic spread^71^. It was used as a single cycle retrograde tracer as described in^72^. Viral starter stocks were a kind gift from K.-K. Conzelmann (Max-von-Pettenkofer Institute). Both viral vectors included the gene of the fluorescent protein mCherry. The tracers were injected in the STN of 21 to 35 day-old C57Bl6 mice. Coordinates from Bregma of the injection were: lateral, −1.2 mm; posterior, −1.4 mm; and depth, −4.95 mm. Since preliminary experiments had shown that none of the 3 reporters labelled the injection site enough to accurately check that the injection was made within the limits of the STN, we injected a 20 nL mixture 1/1 of retrograde tracer and of anterograde viral reporter solutions. The anterograde reporters were rAAV2/1-hSynapsin-mCherry-WPRE-hGHa and rAAV2/1-hSynapsin-EYFP-WPRE-hGH for Fluoro-Gold and viral expression vectors, respectively; see below for details on the anterograde reporters. This procedure established that the injection resulted in anterograde expression within the limits of the STN in 5 mice. Our results come from 10 Fluoro-Gold-, 2 CAV-2-, and 5 RabV- labelled neurons.

### Identification and stimulation of Cx-STN fibers in the STN

We transfected the motor cortex using anterograde expression vectors. Adenoviruses carrying the gene of a fluorescent reporter protein (either mCherry or YFP) or fusion genes coding for ChR2 and one of the fluorescent reporter proteins were injected unilaterally into the cortex of C57Bl6 and D5^−/−^ 21- to28-day-old mice. Viral solutions (titer ≈ 10^12^ IU/mL) were purchased from the Vector Cores of the Universities of Pennsylvania or North Carolina. Viral DNA were: rAAV2/1-hSynapsin-hChR2(H134R)-EYFP-WPRE-hGH and rAAV2/1-hSynapsin-hChR2(H134R)-mCherry-WPRE-hGH and their control counterparts (i.e.: rAAV2/1-hSynapsin-EYFP-WPRE-hGH or rAAV2/1-hSynapsin-mCherry-WPRE-hGH). Injection volumes were 0.5 μL and 3 injections were made. Motor cortex was targeted using coordinates derived from^73^ and iteratively adjusted for smaller animals in initial experiments. Typical coordinates from Bregma were: lateral, −1.125/−1.125/−1.375 mm; posterior, +1.4/+1.15/+1.4 mm; and depth, −1.275/−1.275/−1.475 mm. Location of the reporter was routinely checked using a mouse brain atlas^74^. In a subset of experiments, we functionally assessed transfection location by recording striatal medium spiny neurons in coronal brain slices. Only medium spiny neurons in the dorsolateral margin of the striatum, close to the corpus callosum, were found to be responding to optical stimulations, as expected for medium spiny neurons in the motor area of the striatum. To test whether ChR2-EYFP expression changed the basic electrical properties of the pyramidal neurons in layer 5 of the motor cortex, whole-cell patch-clamp recordings were performed on brain slices obtained from naive and virally-transfected mice. They did not reveal any significant differences in the intrinsic membrane or firing properties of ChR2-positive and ChR2-negative neurons. Furthermore, there was no significant difference between the input-output curves of discharge frequency *versus* injected current (p > 0.05, non-parametric variance analysis and marginal regression, n = 28, 14 neurons in each group; data not shown).

### Slice preparation

After allowing 1 week for Fluoro-Gold diffusion or 2–3 weeks for viral expression, or in 35–50-day old control non-injected animals, acute parasagittal slices were made. Animals were deeply anesthetized and a saturated (95% O_2_/5% CO_2_), ice-cold solution containing (in mM): 250 sucrose, 26 NaHCO_3_, 7 MgSO_4_, 2 KCl, 1.15 NaH_2_PO_4_, 0.5 CaCl_2_, and 11 glucose (pH 7.35) was perfused intracardially before the brain was quickly removed and cut into 350 μm-thick slices using a VT-1200S vibratome (Leica). The slices were transferred in a storage chamber for 40 min at 32 °C. The storage solution was an artificial cerebral spinal solution (referred to as ‘saline’, composed of (in mM): 124 NaCl, 26 NaHCO_3_, 3.6 KCl, 1.3 MgCl_2_, 2.4 CaCl_2_, 1.25 HEPES, 10 glucose at pH 7.35, and saturated by bubbling 95% O_2_/5% CO_2_) supplemented with 5 μM glutathion and 1 mM sodium pyruvate. Slices were then maintained at room temperature in the same solution until used for recording.

### Histology

Immunohistochemistry was performed on 350 µm-thick slices previously used for electrophysiology or on 50 µm-thick brain slices obtained from perfusion-fixed animals as previously described^75^. Following recording, the slices were fixed overnight in 4% paraformaldehyde in 0.1 M phosphate buffer (PB), and then kept until used in PB supplemented with 0.2% sodium azide at 4 °C. After 1 h of incubation in “Triton PBS” (PBS with 0.3% v/v Triton X-100 and 0.02% w/v sodium azide; Sigma) containing 1% v/v BSA (Jackson ImmunoResearch Laboratories), sections were incubated overnight in Triton PBS containing 1% v/v BSA and the appropriate mixture of two primary antibodies: goat anti-forkhead box protein P2 (FoxP2; 1:200; Santa Cruz Biotechnology, sc-21069); rabbit anti-Fluoro-Gold (1:20 000; Millipore, AB153); rat anti-red fluorescent protein (1:1000; Chromotek, 5F8 αred); rabbit anti-yellow fluorescent protein (1:1000, Invitrogen, A11122). This step was at room temperature except for labelling Fluoro-Gold which was made at 4 °C. After exposure to primary antibodies, slices were washed in PBS and incubated at room temperature in Triton PBS for 2 hours containing a mixture of the appropriate secondary antibodies: donkey anti-rat Alexa 594 (1:500; Invitrogen, A 21 209); donkey anti-goat Alexa 488 (1:500; Life Technology, A11 055); donkey anti-rabbit Alexa 488 (1:1000; Life Technology, AV 21 206). Finally, after washing in PBS, all slices were mounted in Vectashield (H-1000 or H-1200, Vector Laboratories, USA). Confocal macroscopy as well as microscopy was used for imaging.

### Electrophysiology

Whole cell patch clamp experiments were made at 32 °C in a submersion recording chamber under an Examiner Z1 (Zeiss, Germany) upright microscope. Slices were bathed in the saline described above (without glutathion and sodium pyruvate), at a rate of 3 mL/min. Whole-cell recording pipettes (4–6 MΩ) contained (in mM) 140 K-gluconate, 11 EGTA, 10 HEPES, 1 CaCl_2_, 2 ATP-Mg, and 0.4 Na-GTP for the current clamp experiments in the cortex. They contained an otherwise similar solution to the exception of K-gluconate, replaced by CsCl, and addition of 2 mM QX314, for the voltage clamp experiments performed in the STN. In the subset of experiments that established the I.V. curve and conductance of AMPA EPSCs, a specific intra-pipette solution was used. Its composition was (in mM): 120 cesium methanesulfonate, 10 NaCl, and 5 TEACl, in addition to 11 EGTA, 10 HEPES, 1 CaCl_2_, 2 ATP-Mg, 0.4 Na-GTP and 2 QX314. In all cases, the osmolarity of intra-pipette solutions was between 280 and 300 mOsm and pH was adjusted to 7.25. Cells were visualized under IR-DIC and fluorescence microscopy. Experiments were conducted with Axon Instruments hardware and software (Axopatch-1D, Digidata 1322 A, and PClamp V9.2; Molecular Devices, USA), with the amplifier filter set at 5 kHz, and the digitization rate at 20 kHz. Voltages were corrected off line for liquid junction potentials.

### Optical stimulations

Optical stimulations were performed using a 473 nm diode pumped solid state laser (Optotronics, USA) connected to a 800 μm optical fiber (Errol, Paris, France) usually positioned at the surface of the slice next to the recording site. We also assessed positions of fiber away from the recording site, in a more frontal area, with light directed to the internal capsule (and not reaching the STN). We found no major change in the responses to light to the exception of their size (which was much smaller) and the frequency of their occurrence (which was considerably reduced). In addition, we tested moving the fiber from the STN area to the internal capsule at more frontal locations while recording the light-evoked responses from a STN neuron. Again, we did not find major changes except their size which progressively diminished until responses most often disappeared. The maximum output power at the end of the fiber was 1.15 mW.mm^−2^.

### Recording protocols

First, neurons were held in the voltage clamp mode at −60 mV, and −5 mV steps were applied for off line determination of intrinsic cell parameters. Then, current clamp or voltage clamp modes were used. Intrinsic properties and firing of the Cx-STN neurons were studied at zero current level by presenting families of currents steps ranging from −200 to +600 pA in 40 pA increments, in saline supplemented with inhibitors of fast synaptic transmission i.e. 20 μM DNQX, 50 μM APV, 50 μM picrotoxin and 1 μM CGP 55845. The activation of Cx-STN transmission from corticofugal axons was performed in voltage clamp mode in the continued presence of GABA A and B receptor antagonists (50 μM picrotoxin and 1 μM CGP 55845) to avoid the occurrence of huge spontaneous inhibitory events. The membrane was held at −80 mV unless otherwise stated. A −5 mV voltage step was included at the beginning of all sweeps to allow off line control of access resistance and cell parameters, and single optic stimulations were applied. Their duration and luminance were adjusted to produce a smooth light-evoked current with half maximal amplitude whenever this was possible. Flash durations were always smaller than or equal to 1 ms. We found that light-evoked AMPA currents usually first increased amplitude and then either markedly run down or maintained amplitude. The neurons showing such a rundown were discarded on line. The phases of stabilization and/or adjustment lasted approximately 20 min and were not analyzed. Single flashes or trains were applied at 20 sec intervals, and repeated between 6 and 12 times. Slices were discarded after trains at 40 Hz, to prevent the possible interference of induction of plasticity.

### Pharmacology

Unless otherwise stated, drugs were diluted in saline and perfused in the same way as control saline. We used SKF 82958, SKF 81297, or SKF 38393, 2–5 µM. The 3 dopaminergic agonists were used to neutralize the differences in the affinity or potency of the drugs for D5 or D1 receptors reported in the literature. The term ‘D1/D5 agonists’ refers to SKF 82958, SKF 81297 and SKF 38393 (2.5 to 5 μM). We found no qualitative difference in the action of the three drugs. Possible quantitative differences between the three drugs were not investigated. In these experiments, the ‘before’ values were the averaged 6–12 values in saline immediately preceding the drug perfusion, ‘after’ referring to the averaged 6–12 values measured after 10 min of perfusion.

### Drugs and chemicals

Unless otherwise stated, drugs were obtained from Tocris Bioscience (UK) or Abcam (France), except SKF-82958, which was purchased from Sigma (France).

### Data analysis

Data were analyzed off line using pClamp V9.2 (Molecular Devices, USA), Origin V7 (OriginLab, USA), Prism5 (GraphPad Software, USA) and R (R Foundation for Statistical Computing, Austria). Access resistance and cell parameters were calculated from the average response to 5 steps of −5 mV in voltage clamp. The firing of Cx-STN neurons was characterized as follows. When the neurons did not fire action potential at rest, resting membrane was the mean voltage at zero current level. The sag index was the mean ratio of the peaks to the steady state voltages induced by the steps from −200 pA to −40 pA. Rheobase was the value of the step inducing the first action potential; it defined the threshold. Input-output was characterized by plotting the firing frequency for all steps, or for those above threshold. In the latter case, the current values represented the amplitude of the step minus the rheobase value. Firing-current slopes (f.i slope) and linearity indexes were derived from the firing frequency above threshold values. F.i slope was the slope of the linear fit of the firing frequency above threshold curve. Linearity index was the slope of the 3^rd^ interval divided by the slope over the 1^st^ interval. The instantaneous firing frequency was measured as a function of time, set in bins of 50 ms, from the onset of the current traces at threshold, or with approximately 30 Hz average firing rates when 30 Hz was not threshold. Spike firing adaptation (SFA) was the ratio of the last two interval interspikes (ISI) to the first 2 ISI. It was calculated at threshold if this elicited at least 5 AP, and for the step that produced approximately a firing rate of 30 Hz. The Cx-STN transmission was studied in the voltage clamp mode. Peak and charge of photo-evoked current, peak of capacitive transient and holding current were detected and plotted against time. Neurons with an initial value of access resistance greater than 35 MΩ or showing a change of access resistance of more than 20% over the recording period were discarded. Values obtained from 6 to 12 consecutive single sweep currents were averaged to be included in any group data. Onset latency of current was measured at current value equal to mean holding current minus 2 SD. The chord conductances were calculated in the same way as^35^. The rectification index (RI) was calculated using chord conductances (g). The following equation was used: g_+40_/g_-80_ = (I_+40_/(40 − Erev)/(I_−80_(80 − Erev). Thus, RI is expressed as a ratio that will decrease when rectification increases. I_−80_ and I_+40_ are the EPSC current amplitudes recorded by holding the membrane potential at −80 mV and +40 mV, respectively. The Erev values were obtained from the I–V plots.

### Statistics

In the text, all values are given as means ± SEM, when mean and median are close. If this is not the case, both median and mean are given. Box plots are used for graphic presentation of electrophysiological data, due to the small sample size. The box plot presents the distribution with the median as a central line. The hinges and edges of the box display the 25^th^ and 75^th^ percentiles, whereas the “whiskers” display the minimal and maximal values. Statistical comparisons were made using non-parametric tests with an α level of 0.05. The Mann and Whitney test, as well as the Wilcoxon signed-rank test, were used for data obtained from independent and matched paired samples, respectively. The Wilcoxon test was also used to assess difference from a hypothetical value.

## Electronic supplementary material

Supplementary Information
