# Supplementary material for: D5 dopamine receptors control glutamatergic AMPA transmission between the motor cortex and subthalamic nucleus
Source: Sci Rep. 2018 Jun 11;8:8858. doi: 10.1038/s41598-018-27195-6 (PMC5995923; doi:10.1038/s41598-018-27195-6)
Supplement: Supplementary file 1 — Supplementary Information [file 41598_2018_27195_MOESM1_ESM.pdf]

D5 dopamine receptors control glutamatergic AMPA transmission between motor cortex and subthalamic nucleus

By Lionel Froux, Morgane Le Bon-Jego, Cristina Miguelez, Elisabeth Normand, Stephanie Morin, Stéphanie Fioramonti, Massimo Barresi, Andreas Frick, Jerome Baufreton and Anne Taupignon

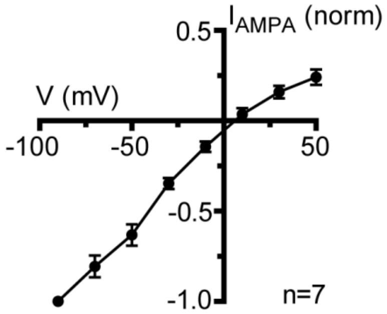

Supplemental Figure S1: Voltage-dependence of pharmacologically-isolated AMPA receptor EPSCs
I-V curves of photo-induced AMPA synaptic currents reversed close to 0 mV and displayed rectification. Mean normalized EPSCs (I_{AMPA}) are plotted against voltage. Holding voltage was changed from – 90 to +50 mV in 20 mV steps. Records in the presence of the specific NMDA receptor antagonist, APV (50 µM).

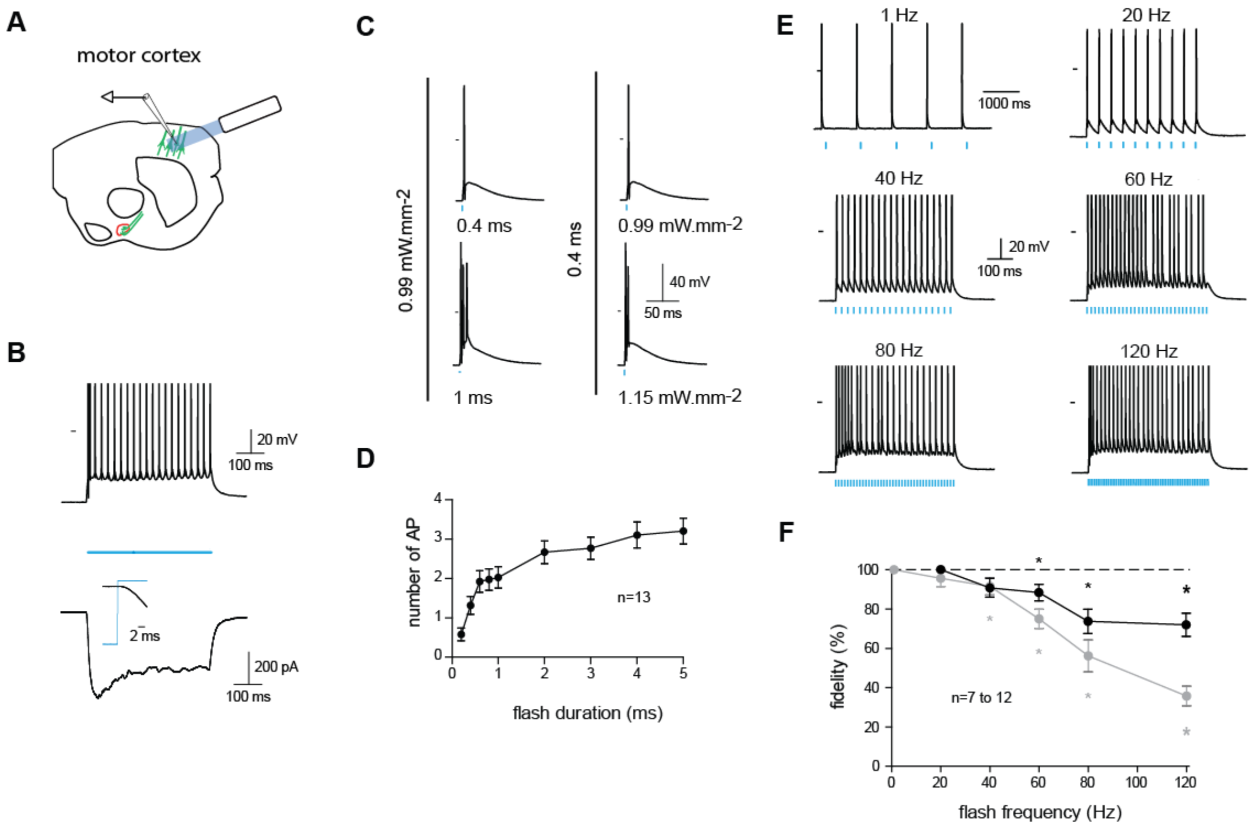

Supplemental Figure S2: Repetitive optical stimulations up to 40 Hz reliably induced repetitive action potential firing in ChR2-expressing pyramidal cortical neurons

A. Schematic of the experimental configuration. Cortex of wild-type mice was injected with a solution of an adenovirus carrying the ChR2-EYFP or ChR2-mcherry fusion protein gene. **B.** Prolonged 473 nm photostimulation (blue bar, middle) directed at the motor cortex generated spikes throughout the stimulation period (top trace) or a biphasic photocurrent with an initial peak followed by a sustained component (bottom trace) in layer 5 pyramidal neurons. The traces were recorded in different neurons. The inset illustrates the short latency to onset of photocurrent (thick trace, photocurrent and thin blue trace, flash). **C.** Example of coordinated changes in flash luminance and duration. Increasing flash duration from 0.4 to 1 ms at a luminance of 0.99 mW.mm^{-2} induced the discharge of 2 additional action potentials. Conversely, increasing the luminance of a 0.4 ms flash from 0.99 mW.mm^{-2} to 1.15 mW.mm^{-2} produced 1 additional action potential. **D.** Summary graph of the mean number of action potentials elicited by increasing flash durations. Luminance range: 0.66 to 1.15 mW.mm^{-2}. **E.** Example of discharges due to trains of 0.3 ms flashes (blue bars) at frequencies between 1 and 120 Hz in a ChR2-EYFP-positive layer 5 pyramidal neuron. Spikes were evoked by each flash in trains between 1 and 40 Hz. Trains of flashes at frequencies higher than 40 Hz were less efficient and the number of spikes was lower than the number of flashes. Flashes luminance was 0.6 mW.mm^{-2}. **F.** Summary data of experiments similar to those illustrated in E. Mean ± SEM are shown. Fidelity is the ratio of spike to flash counts, expressed in %. 1 was registered for one action potential for one flash; zero for no action potential, as well as two or more action potentials. Fidelity was calculated for the first 8th flashes or the entire 500 ms train (black and gray curves, respectively). In the 1 to 40 Hz range, each of the first 8 flashes reliably triggered a single action potential. Later flashes in the 40 Hz train or at higher flash frequencies sometimes failed to trigger action potentials.

* indicates value significantly different from 1, α= 0.05, two-tailed Wilcoxon Signed Rank Test

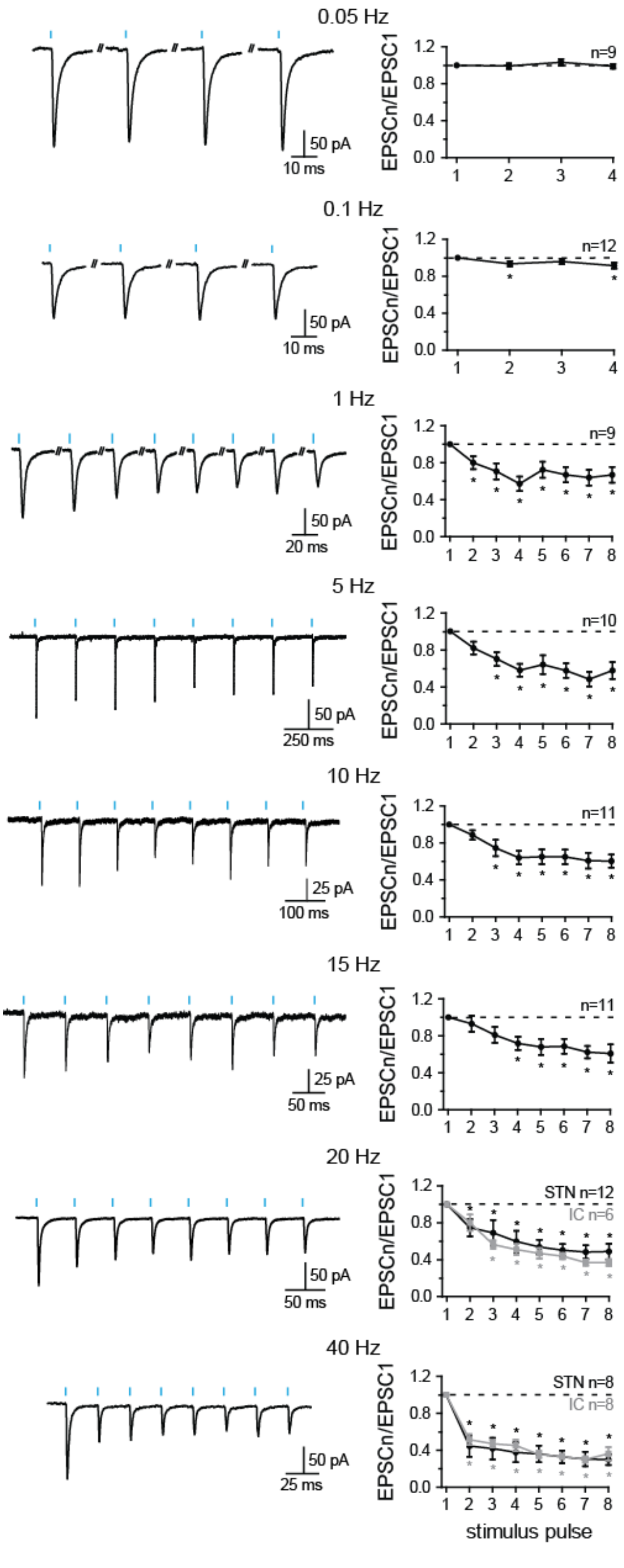

Supplemental Figure S3: Short-term plasticity of the cortico-subthalamic synapses at light frequencies between 0.05 and 40 Hz
EPSCs were induced by repetitive trains of flashes. Representative examples of EPSCs obtained at light frequencies of 0.05, 0.1, 1, 5, 10, 15, 20 and 40 Hz (left) and group values (right). All trains contained 8 photo-stimuli, except those at 0.05 and 0.1 Hz. At theses low frequencies, trains comprised only four photo-stimuli. Mean response amplitudes normalized to the first responses were plotted as a function of stimulus number within trains. The gray symbols of the 20 and 40 Hz graphs depict the values obtained by flashing the internal capsule (and not the STN)
* values significantly different from 1, α= 0.05, two-tailed Wilcoxon Signed Rank Test
